# The Role of Non-Statin Lipid Lowering Therapies to Reduce ASCVD Events in Primary Prevention

**DOI:** 10.1007/s11883-025-01283-1

**Published:** 2025-04-02

**Authors:** Chukwuemezie Kamanu, Dean G. Karalis

**Affiliations:** Department of Cardiology, Jefferson University Hospital, Sidney Kimmel Medical College, 227 North Broad Street, Suite 200, Philadelphia, PA 19107 USA

**Keywords:** Primary prevention, Non-statin therapies, Lipid-lowering therapies, LDL cholesterol reduction

## Abstract

**Purpose of Review:**

Atherosclerotic cardiovascular disease (ASCVD) remains a leading global health challenge, with low-density lipoprotein (LDL) cholesterol a pivotal risk factor. While statins are cornerstone therapy for lowering LDL cholesterol, many high-risk primary prevention patients are unable to tolerate statin therapy and do not achieve their guideline directed LDL cholesterol goal. For these patients, non-statin therapies offer complementary and alternative approaches to LDL cholesterol reduction.

**Recent Findings:**

Recent advancements in non-statin therapies have expanded the options available to clinicians to lower LDL cholesterol in high-risk primary prevention patients. Yet these medications are often under-utilized in clinical practice.

**Summary:**

Observational studies, Mendelian randomization studies, and randomized clinical trials support the role of non-statin LDL cholesterol lowering therapies in the primary prevention of ASCVD. This review summarizes the evidence supporting their use for the primary prevention of ASCVD and offers practical suggestions as to how clinicians can integrate these medications into their clinical practice.

## Introduction

Atherosclerotic cardiovascular disease (ASCVD) is the leading cause of morbidity and death for both men and women in the United States. Due to advances in the diagnosis and treatment of ASCVD, death rates from cardiovascular (CV) disease have declined steadily over decades, however over the last decade this trend has reversed [1]. The national CV death rate declined by 8.9% from 2010 to 2019 and then increased by 9.3% from 2019 to 2022. Younger adults aged 35 to 54 and Black adults had the greatest increase in CV deaths [2]. For this reason, there has been an enhanced focus on primary prevention.

Atherosclerosis is a progressive process driven by inflammation and deposition of atherogenic apolipoprotein B containing particles within the intima of coronary arteries, of which low-density lipoprotein (LDL) particles are most prevalent. Statins lower LDL and other apolipoprotein B containing particles and randomized clinical trials with statin therapy have shown significant reductions in CV events in both primary and secondary prevention [3]. Since atherosclerosis develops over decades there is a rationale to start lipid lowering therapy early which may reduce CV risk more than starting it later after an individual has had a CV event. Both national and international societies have provided guidelines for cholesterol management for the primary prevention of CV disease. The 2018 AHA/ACC/Multisociety cholesterol guideline identified several patient groups without ASCVD who would benefit from statin therapy [4]. They include patients with severe hypercholesterolemia (LDL cholesterol *≥* 190 mg/dL) and those 40 to 75 years of age with diabetes or whose 10-year ASCVD risk is high.

There is now robust evidence from multiple randomized clinical trials that statin therapy reduces CV risk in high-risk primary prevention patients and for these patients, guidelines recommend moderate to high-intensity statin therapy. However, up to 30% of patients discontinue their statin or take a lower than guideline directed dose due to statin intolerance [5]. Statin intolerance has important clinical implications. Patients on a less than recommended statin dose or those who discontinue their statin are at increased risk of a CV event [6]. Findings from randomized controlled trials of non-statin therapies and Mendelian randomization studies provide evidence to support the use of non-statin therapies to reduce ASCVD risk in patients who do not achieve their guideline-directed LDL cholesterol goal despite lifestyle and maximally tolerated statin therapy [7]. However, these agents are under-utilized in clinical practice [8].

There are now several available non-statin therapies that lower LDL cholesterol and other atherogenic lipoproteins. They include ezetimibe, bile acid sequestrants, PCSK9 inhibitors, bempedoic acid, fibrates, icosapent ethyl, and niacin. Recent guidelines have provided recommendations for adding non-statin lipid-lowering agents to patients with primary hyperlipidemia or those at high-risk for ASCVD when further LDL cholesterol lowering is needed [9, 10]. These guidelines emphasize the use of ezetimibe for add-on therapy but provide less guidance for the use of other non-statin therapies. Since the publication of these guidelines newer information on the use of these agents has led the FDA to approve several other non-statin agents as add-on therapy to lifestyle and maximally tolerated statin therapy to reduce ASCVD risk in high-risk primary prevention patients. This review examines the clinical evidence for the use of non-statin therapy for the primary prevention of CV disease, reviews their safety, and provides practical clinical suggestions on how to better assess CV risk in adults without ASCVD who would benefit from further lowering of LDL cholesterol with these agents.

### Ezetimibe

Ezetimibe acts as a selective cholesterol absorption inhibitor, targeting uptake within the jejunal enterocyte brush border. Its primary mechanism of action involves binding to the Niemann-Pick C1-like 1 protein, a key cholesterol transport protein. This targeted interaction disrupts intestinal cholesterol absorption, leading to a compensatory increase in hepatic LDL receptor synthesis. Consequently, ezetimibe promotes enhanced clearance of LDL cholesterol from the circulation, resulting in a net reduction in serum LDL cholesterol levels [11]. Clinical studies have shown that ezetimibe is well tolerated and reduces LDL cholesterol by 18% when used as monotherapy and up to an additional 25% when combined with statin therapy [9].

Ezetimibe has been shown to reduce CV events when added to statin therapy in patients with a recent acute coronary syndrome [12]. Although there is less evidence for its use in primary prevention, Mendelian randomization trials and 2 clinical studies suggest that ezetimibe can reduce CV events in high-risk primary prevention patients. The SHARP (Study of Heart and Renal Protection) trial investigated the efficacy of ezetimibe-statin combination therapy compared to placebo in 9270 patients with chronic kidney disease [13]. The study population achieved a significant reduction in LDL cholesterol levels (approximately 32 mg/dL). This resulted in a 17% relative reduction in major CV events over a median follow-up of five years. The findings also demonstrated a lower incidence of a composite endpoint of myocardial infarction, coronary death, ischemic stroke, or revascularization in the ezetimibe-statin group compared to placebo (11.3% vs. 13.4%, hazard ratio 0.83, 95% CI 0.74–0.94, *p* = 0.0021). The combination of statin and ezetimibe was well tolerated. The trial did not include an ezetimibe only arm making it difficult to assess the added benefit of adding ezetimibe to statin therapy. However, the benefit of ezetimibe alone in primary prevention was investigated in a trial in Japan. The EWTOPIA-75 trial assessed the efficacy of ezetimibe without statin therapy for reducing ASCVD events in 3796 patients 75 years of age or older who had no history of CV disease [14]. All participants received dietary counseling and were randomized to receive either ezetimibe or usual care. Over a median follow-up of 4.1 years, ezetimibe significantly reduced the incidence of the primary outcome (composite of sudden cardiac death, myocardial infarction, coronary revascularization, or stroke) by 34% (hazard ratio 0.66, 95% CI, 0.50–0.86; *p* = 0.002). Regarding the secondary outcomes, the incidence of a composite of cardiac events (hazard ratio, 0.60; 95% CI, 0.37–0.98; *p* = 0.039) and coronary revascularization (hazard ration, 0.38, 95% CI, 0.18–0.79; *p* = 0.007) were lower in the ezetimibe group compared to the control group.

### Monoclonal Proprotein-Convertase Subtilisin/Kexin Type 9 Inhibitors

In the liver, hepatocytes synthesize and secrete proprotein convertase subtilisin/kexin type 9 (PCSK9). Circulating PCSK9 binds to LDL receptors on the surface of the hepatocyte, initiating a pathway that leads to their degradation within lysosomes reducing the clearance of LDL particles from the bloodstream [15]. Several therapeutic approaches have emerged that inhibit PCSK9, primarily utilizing monoclonal antibodies. These therapies function by decreasing circulating PCSK9 levels. Consequently, with less PCSK9 available to bind to LDL receptors an upregulation of LDL receptors on the surface of the hepatocyte leads to improved clearance of LDL particles from circulation reducing circulating LDL cholesterol levels [10]. Currently, the only approved monoclonal PCSK9 inhibitors are evolocumab and alirocumab. Evolocumab (140 mg/mL) is self-administered subcutaneously every 2 weeks via a prefilled syringe. The prefilled syringe contains latex and is contraindicated in patients with a latex allergy and for patients with a latex allergy a monthly dose of 420 mg/mL administer via an autoinjector is available. Alirocumab is available in 2 doses, 75 mg or 150 mg self-administered subcutaneously every 2 weeks. Phase 3 trials have shown that similar doses of evolocumab and alirocumab when added to high-intensity or maximally tolerated statin therapy significantly reduced LDL cholesterol from baseline by up to 62%, non-high-density lipoprotein (HDL) by up to 52%, and apolipoprotein B by up to 53% at 24 weeks. Triglycerides were modestly lowered, HDL cholesterol increased by a small amount, and lipoprotein(a) was lowered by up to 25 to 30%. Overall, the monoclonal PCSK9 inhibitors are well tolerated. Side effects include local site reactions and nasopharyngitis which are usually mild and self-limited [16, 17]. Adult patients with severe hypercholesterolemia defined as an LDL cholesterol *≥* 190 mg/dL but without clinical ASCVD are at very high lifetime CV risk. The very high cholesterol in these patients is often genetically determined and most patients will have either polygenic or monogenic familial hypercholesterolemia (FH). Current guidelines recommend consideration of a PCSK9 inhibitor as add-on non-statin therapy for these patients if they have had < 50% reduction in LDL cholesterol or their LDL cholesterol is *≥* 100 mg/dL despite maximally tolerated statin therapy and lifestyle changes [9]. Both evolocumab and alirocumab have been approved by the FDA for use in primary hyperlipidemia as well in heterozygous and homozygous FH.

While clinical outcome studies with PCSK9 inhibitors have focused on secondary prevention of ASCVD, recent evidence suggests a potential role in primary prevention as well. The SPIRE I and SPIRE II trials enrolled a total of 27,438 patients with high ASCVD risk. Inclusion criteria included either a prior ASCVD event or a high risk of future ASCVD without a prior event [18]. All participants were receiving standard treatment with high-intensity or maximally tolerated statin therapy. Baseline LDL cholesterol levels were required to be at least 70 mg/dL in the SPIRE-1 and 100 mg/dL in the SPIRE-2 trials. Participants were administered the humanized monoclonal PCSK9 inhibitor bococizumab 150 mg subcutaneously every 2 weeks. Compared to the placebo group, bococizumab reduced LDL cholesterol by 54% at week 12, however the reduction was attenuated to 43% by 52 weeks. The attenuation in LDL cholesterol reduction was greater in patients who developed antidrug antibodies. In the SPIRE-1 outcome study of patients with an LDL cholesterol > 70 mg/dL at 7 months there was no difference in the primary endpoint of a composite of major adverse CV events including non-fatal myocardial infarction and stroke, hospitalization for unstable angina requiring urgent revascularization, or CV death. However, in the SPIRE-2 study of patients with an LDL cholesterol > 100 mg/dL at 12 months there was a significant reduction of 21% in the primary endpoint (hazard ratio 0.79, 95% CI, 0.65–0.97). Due to the high prevalence of antidrug antibodies and the attenuation of LDL cholesterol over time drug development was stopped and the drug never made it into the market. However, the findings from this study suggest that PCSK9 inhibitors may provide benefit in a high-risk primary prevention patient population. These findings are supported by emerging evidence that LDL cholesterol reduction with PCSK9 inhibitors leads to plaque regression and stabilization when added to statin therapy [19].

A randomized controlled clinical trial is presently investigating the direct impact of PCSK9 inhibitors on CV outcomes in a population free from ASCVD. The VESALIUS-CV trial is a global, randomized, double-blind, placebo-controlled study designed to assess the impact of evolocumab on major adverse cardiovascular events in high-risk patients without prior ischemic events [20]. The study enrolled 12,301 participants with either established atherosclerosis or high-risk diabetes, but no prior myocardial infarction or stroke with a baseline LDL cholesterol ≥ 90 mg/dL, non-HDL cholesterol ≥ 120 mg/dL, or apolipoprotein B ≥ 80 mg/dL, on optimized lipid-lowering therapy. Randomization was conducted in a 1:1 ratio, assigning participants to either evolocumab 140 mg administered subcutaneously every 2 weeks or a matching placebo. The primary efficacy objective is to determine if evolocumab reduces the incidence of the co-primary composite endpoint of either coronary heart death, myocardial infarction, or ischemic stroke, as well as the broader endpoint encompassing coronary heart death, myocardial infarction, ischemic stroke, or ischemia-driven revascularization procedures. Results are expected in the next several years.

### Inclisiran

Building upon the success of monoclonal antibodies for PCSK9 inhibition, recent research has explored siRNA-based therapies for lowering LDL-C. These siRNA molecules leverage the endogenous RNA interference (RNAi) pathway to target and degrade PCSK9 mRNA. This degradation process leads to a reduction in PCSK9 protein production offering a novel therapeutic approach for dyslipidemia management. The only FDA approved siRNA targeting PCSK-9 is inclisiran [21]. Inclisiran exhibits a unique biannual dosing regimen, with an initial subcutaneous injection followed by a second dose at 3 months. Thereafter, maintenance therapy is achieved with subsequent injections every 6 months. Two phase 3 placebo-controlled trial, ORION-10 and ORION-11 assessed the efficacy and safety of inclisiran in patients at high-risk for ASCVD whose LDL cholesterol was elevated despite maximally tolerated statin therapy. In total 3178 patients were randomized to inclisiran (284 mg) or placebo, administered subcutaneously on day 1, day 90, and every 6 months thereafter for a period of 540 days. Inclisiran therapy achieved an approximate 50% reduction in LDL cholesterol and was overall well tolerated. As with the monoclonal PCSK9 inhibitors, more injection site reactions occurred with inclisiran compared to placebo [22]. Based on the findings from these studies the FDA approved inclisiran as an adjunct to diet and maximally tolerated statin therapy for the treatment of adults with clinical ASCVD or heterozygous FH who need additional LDL cholesterol lowering.

Inclisiran’s therapeutic focus has primarily been on secondary prevention of ASCVD. However, data from the pre-specified analysis of the ORION-11 trial suggests benefits in primary prevention. This analysis included 203 individuals at high risk for ASCVD, but without prior CV events. Despite receiving maximally tolerated statin therapy, these individuals had elevated LDL cholesterol levels. Inclisiran treatment in this subgroup demonstrated significant reductions in LDL cholesterol compared to placebo, with a placebo-corrected decrease of 43.7% at day 510 [23]. Considering the successful results of the ORION-11 trial, the FDA granted an expanded approval for inclisiran in July 2023. This approval extends inclisiran’s use to high-risk primary prevention patients, including those with heterozygous FH, type 2 diabetes, or whose 10-year ASCVD risk exceeds 20%, and who require additional LDL cholesterol reduction despite maximally tolerated statin therapy [24].

The ongoing, randomized controlled trial ORION-4 (NCT03705234), with an estimated completion date of 2026, is investigating the impact of inclisiran on major adverse cardiovascular events (MACE). The study is recruiting approximately 15,000 participants with pre-existing atherosclerotic cardiovascular disease. Men aged 40 years or older and women aged 55 years or older will be eligible. In a 1:1 randomization scheme, participants will receive either inclisiran sodium 300 mg or a matching placebo administered subcutaneously on three occasions: at baseline, 3 months, and then every 6 months for a planned median follow-up duration of 5 years [25]. VICTORION-1-PREVENT is an ongoing, randomized, placebo-controlled trial enrolling approximately 14,000 participants designed to assess the efficacy and safety of inclisiran 300 mg administered subcutaneously in reducing the risk of major CV events in adult patients at high ASCVD risk. Participants are randomly assigned to receive either inclisiran or placebo subcutaneously on day 1, day 90, and every six months thereafter. As an event-driven study, data collection will continue until a predetermined number of clinical events occur in both treatment arms, with a minimum three-year follow-up for all participants during the double-blind period [26].

For some Medicare patients the cost of the monoclonal PCSK9 inhibitors is often too high. Inclisiran is unique among other lipid-lowering therapies in that it must be administered by a healthcare professional through a subcutaneous injection. Clinicians or hospital systems are required to buy the product and bill Medicare under Part B, or they may refer patients to independent alternate injection centers (AIC) who complete the preauthorization process and administer the medication. In an early experience report of 37 patients prescribed inclisiran in an academic lipid clinic, all Medicare patients were able to obtain access to inclisiran compared with only 25% of patients with non-Medicare insurance [27]. In addition, more than one-half of Medicare patients were able to obtain the drug without preauthorization. For Medicare patients in whom the monoclonal PCSK9 inhibitors are denied or too expensive, inclisiran is a practical alternative, though CV outcome trials are still ongoing to confirm if it can provide similar outcomes as seen with the monoclonal PCSK9 inhibitors.

### Bempedoic Acid

Bempedoic acid is a novel oral cholesterol-lowering medication that acts by inhibiting ATP-citrate lyase. This enzyme lies upstream of HMG-CoA reductase, the target of statins, within the cholesterol biosynthesis pathway. Like statins, bempedoic acid’s inhibitory action promotes upregulation of LDL receptor expression, leading to enhanced clearance of LDL particles from the circulation [9, 28]. Importantly, bempedoic acid requires hepatic activation, lacking conversion in skeletal muscle. This distinct mechanism translates to a significantly lower risk of statin-associated muscle symptoms compared with statin therapy [9, 28].

The CLEAR (Cholesterol Lowering via Bempedoic Acid [ECT1002], and ACL-Inhibiting Regimen) Outcomes trial investigated the efficacy and safety of bempedoic acid in statin-intolerant patients who had or were at high ASCVD risk. A total of 13,970 participants who were unable to tolerate more than a very low dose of a statin were randomized to receive oral bempedoic acid (180 mg daily) or placebo. The mean age of the patients was 65 years of age, almost half were female, and 30% were primary prevention. At baseline, 22.7% of patients were on low-dose statins and 11.5% on ezetimibe. Bempedoic acid achieved a mean 21% reduction in LDL cholesterol compared to placebo. With a median follow-up of 40.6 months, the bempedoic acid group demonstrated a statistically significant decrease in the primary composite endpoint (CV death, nonfatal myocardial infarction, nonfatal stroke, or coronary revascularization) compared to placebo (11.7% vs. 13.3%, hazard ratio 0.87, 95% CI 0.79–0.96, *p* = 0.006, absolute risk reduction 1.6%) [29]. Among the key secondary endpoints non-fatal myocardial infarction was reduced by 27% (hazard ratio 0.73, 95% CI 0.62-0.087) and coronary revascularization by 19% (hazard ratio 0.81, 95% CI 0.72–0.92). At 6 months, LDL cholesterol was lower in the bempedoic acid group by 20.3%; however, this effect was less towards the end of the trial, with a 15.9% greater LDL cholesterol reduction in the bempedoic acid group compared to placebo. Notably, a higher proportion of patients in the placebo group (15.6%) initiated additional lipid-lowering therapy during the trial compared to the bempedoic acid group (9.4%). This difference in treatment intensification may partially explain the attenuation of the LDL cholesterol lowering gap between the groups over time and may have lessened the difference in outcomes between the 2 groups [29].

The CLEAR-Outcomes trial enrolled over 4000 high-risk primary prevention patients to either bempedoic acid or placebo. In a pre-specified subgroup analysis of these primary prevention patients, bempedoic acid significantly reduced LDL cholesterol by 21.3%. At a median follow-up of 39.9 months, the incidence of the composite primary endpoint (CV death, nonfatal myocardial infarction, nonfatal stroke, or coronary revascularization) was lower in the bempedoic acid group (5.3%) compared to placebo (7.6%), (hazard ratio 0.68, 95% CI 0.53–1.87) [30]. This translates to an absolute risk reduction of 2.3% and a number needed to treat of 43. Overall bempedoic acid was well tolerated though the incidences of gout (3.1% vs. 2.1%) and cholelithiasis (2.2% vs. 1.2%) were higher with bempedoic acid than with placebo. In addition, treatment with bempedoic acid led to small increases in serum creatinine, uric acid, and hepatic enzyme levels [29]. Bempedoic acid is a weak inhibitor of OAT2 (organic ionic transporter 2) which medicates the renal excretion of uric acid. Bempedoic acid should be used with caution in patients with hyperuricemia or those with a history of gout.

The CLEAR Outcomes trial demonstrated the value of bempedoic acid in reducing CV risk in high-risk primary prevention patients who are intolerant to statins. Adding bempedoic acid to a moderate or high-intensity statin will further reduce LDL cholesterol by up to 18% [31], however whether this will further reduce CV risk has not been investigated in patients on higher statin doses. A post-hoc analysis of the CLEAR Outcomes trial investigated whether bempedoic acid’s LDL cholesterol lowering effect translates to similar cardiovascular benefits as statins [32]. The investigators compared CV outcomes for each 1 mmol/L (38.7 mg/dL) of LDL cholesterol reduction with bempedoic acid from CLEAR Outcomes and statins from previously published data from the Cholesterol Treatment Trialists’ Collaboration (CTTC) meta-analysis. For each 1 mmol/L difference in LDL cholesterol the risk of a major CV event was reduced by 25% (hazard ratio 0.75. 95% CI 0.63–0.90) with bempedoic acid and 22% (hazard ratio 0.78, 95% CI 0.76–0.80) with statins. The findings suggest for patients at risk of a CV event, the goal should be to use a combination of medications that are well tolerated to achieve the patient’s optimal LDL cholesterol goal. In this regard, bempedoic acid is also commercially available as a fixed-dose combination with ezetimibe (180 mg bempedoic acid combined with 10 mg ezetimibe). A phase 3 trial demonstrated that after 12 weeks of treatment, this combination therapy achieved a significantly greater reduction in LDL-C levels (36.2%) compared to either bempedoic acid (17.2%) or ezetimibe (23.2%) monotherapy. This combination may offer a more robust LDL cholesterol lowering strategy for patients requiring additional cholesterol management [33].

### Bile Acid Sequestrants

Bile acid sequestrants are a class of cholesterol-lowering medications that act within the intestine. These non-absorbed polymers bind bile acids, hindering their reabsorption and consequently reducing the overall bile acid pool. This triggers a compensatory upregulation of cholesterol conversion into bile acids by the liver, ultimately leading to a decrease in circulating LDL cholesterol levels [34]. At maximum tolerated doses, bile acid sequestrants can achieve moderate reductions in LDL-C levels, ranging from 15 to 25% [9].

The Lipid Research Clinics Coronary Primary Prevention Trial (LRC-CPPT) demonstrated the efficacy of cholesterol lowering in reducing coronary heart disease risk. This randomized, double-blind trial investigated the effects of cholestyramine in asymptomatic men with primary hypercholesterolemia (LDL cholesterol *≥* 190 mg/dL). Over an average follow-up of 7.4 years, 3,806 participants were assigned to receive either cholestyramine or placebo. The study found a significant reduction in the cumulative incidence of the primary endpoint (defined as definite coronary heart disease death and/or definite nonfatal myocardial infarction). The cholestyramine group experienced a 7% event rate compared to 8.6% in the placebo group (hazard ratio 0.81, 95% CI, *p* < 0.05) [35]. Bile acid sequestrant use is hampered by its gastrointestinal side effects, limited ASCVD outcomes data with statins, inconvenient dosing schedules, high pill burden, and potential drug interactions. Bile acid sequestrants can cause severe hypertriglyceridemia when fasting triglycerides are elevated and should not be used when triglycerides are *≥* 300 mg/dL [4]. The emergence of newer non-statin therapies, and the unfavorable side effect profile of bile acid sequestrants, have relegated bile acid sequestrants to a less prominent role for lowering LDL cholesterols. However bile acid sequestrants are considered the only safe lipid lowering therapy during lactation and pregnancy [9].

### Fibrates, Prescription Omega-3 Fatty Acids, and Nicotinic Acid

Fibrates are peroxisome proliferator activated receptor (PPAR)-alpha modulators that modestly lower LDL cholesterol by 5 to 15% [5, 34]. They have a larger effect on triglycerides with reductions of 25 to 35% in patients with high triglycerides (150 to 499 mg/dL) and even greater reductions of up to 55% when triglycerides are severely elevated (*≥* 500 mg/dL) [36]. Two early trials of gemfibrozil monotherapy, the Helsinki Heart Study (HHS) and the Veterans Affairs-HDL Intervention Trial (VA-HIT) showed benefit in reducing CV events compared to placebo [37, 38]. However, two later trials with fenofibrate, Fenofibrate Intervention and Event Lowering in Diabetes (FIELD) and Action to Control Cardiovascular Risk in Diabetes (ACCORD), failed to show any benefit of fenofibrate in reducing CV risk compared to placebo [39, 40]. These early fibrate trials did not specifically enroll patients with elevated triglycerides and subgroup analysis from these trials suggested a benefit of fibrate therapy in patients with elevated triglycerides and low HDL cholesterol. To address this issue the Pemafibrate to Reduce Cardiovascular Outcomes by Reducing Triglycerides in Patients with Diabetes (PROMINENT) trial enrolled 10,497 statin treated patients with type 2 diabetes (of which one-third were primary prevention) with triglycerides between 200 and 499 mg/dL and an HDL cholesterol of 40 mg/dL or less to pemafibrate or placebo [41]. Although Pemafibrate lowered triglycerides by 26.2% and increased HDL cholesterol by 5.1%, it did not reduce the level of apolipoprotein B containing lipoproteins nor the primary endpoint of a composite of CV events or death. The findings from this trial suggest that fibrates should not be used to reduce ASCVD risk among statin treated patients with elevated triglycerides, though they may still have a role to reduce the risk of pancreatitis in patients with severe hypertriglyceridemia.

Two formulations of prescription omega-3 fatty acids are clinically available in the United States. They include omega-3 ethyl ester which is a combination of eicosapentaenoic (EPA) and docosahexaenoic acid (DHA) ethyl ester and icosapent ethyl (IPE) which is a pure ethyl ester formulation of only EPA [42]. Both are approved to treat severe hypertriglyceridemia (*≥* 500 mg/dL), however only IPE is approved to reduce ASCVD risk in high risk primary and secondary prevention patients [43]. The Reduction of Cardiovascular Events with Icosapent Ethyl Intervention Trial (REDUCE-IT) evaluated the efficacy of IPE in reducing cardiovascular risk. A total of 8,179 patients with ASCVD or diabetes (30% of patients) and other risk factors who on statin therapy had a fasting triglyceride level between 135 and 499 mg/dL and an LDL cholesterol *≤* 100 mg/dL were randomly assigned to receive either 2 g of IPE twice daily (total daily dose of 4 g) or a placebo. Over a median follow up of 4.9 years, the primary endpoint, a composite of CV death, nonfatal myocardial infarction, nonfatal stroke, coronary revascularization, or unstable angina, occurred significantly less frequently in the IPE (17.2%) compared to the placebo group (22.0%). This translated to a 25% relative risk reduction (hazard ratio 0.75, 95% confidence interval 0.68–0.83; *p* < 0.001), highlighting the potential CV benefit of IPE in this high-risk primary and secondary prevention population [44]. The Long-Term Outcomes Study to Assess Statin Residual Risk Reduction with Epanova in High CV Risk patients with hypertriglyceridemia (STRENGTH) randomized 13,078 statin-treated patients at high CV risk to a carboxylic acid omega-3 preparation which was a mixture of EPA and DHA at dose of 4 g per day. The trial was prematurely terminated after 39 months due to futility [45]. The negative findings of STRENGTH are in direct contrast with the positive findings in REDUCE-IT. Mineral oil was used as the comparator in REDUCE-IT while corn oil was used in STRENGTH and some have suggested that the mineral oil in the placebo arm of REDUCE-IT may have led to an adverse effect on CV risk. The difference in outcomes between these studies cannot fully be explained by any possible increased risk from mineral oil and it is more likely that the different outcomes are explained by the different doses of EPA used in the 2 trials. Only IPE is approved by the FDA and recommended by current guidelines to lower ASCVD risk in high-risk primary prevention patients whose triglycerides remain elevated on maximally tolerated statin therapy [9].

Nicotinic acid or niacin can lower LDL cholesterol by 5 to 25%, reduce triglycerides by up to 50%, raise HDL cholesterol by up to 15 to 35% and lower lipoprotein (a) by 25% [5, 46]. Although early trials before the era of statin therapy suggested a role for nicotinic acid in reducing ASCVD events, more recent trials in the era of statins failed to demonstrate any benefit of niacin in reducing ASCVD risk when added to statin therapy [47]. Due to the significant side effects of niacin, this medication is no longer recommended for clinical use to treat hyperlipidemia in at risk patients.

## Conclusions

The use of statins to lower LDL cholesterol remains the foundation for the primary prevention of ASCVD. However, not all patients can tolerate statin therapy, and many individuals may not achieve their optimal LDL cholesterol goals and remain at high CV risk. In these cases, alternative LDL cholesterol lowering medications can be considered as an adjunctive treatment to lower LDL cholesterol. The 2018 AHA/ACC/Multisociety cholesterol guideline identified 3 groups of patients who would benefit from statin therapy; severe primary hyperlipidemia (LDL cholesterol *≥* 190 mg/dL), patients aged 40 to 75 with diabetes or whose 10-year ASCVD risk *≥* 7.5%. For these patients, most guidelines identify an LDL cholesterol threshold of *≥* 100 mg/dL on a maximally tolerate statin as to when to consider non-statin LDL cholesterol therapy [4, 9, 33, 36]. For very-high risk primary prevention patients a lower LDL cholesterol threshold is recommended for non-statin add-on therapy. For patients with diabetes and without ASCVD aged 40 to 75 years, the ACC 2022 Expert Consensus Decision Pathway (ECDP) on the role on non-statin LDL cholesterol lowering therapy recommended adding ezetimibe if the 10-year ASCVD risk *≥* 20%, the LDL cholesterol was lowered < 50% and was *≥* 70 mg/dL [9]. The American College of Endocrinology/American Association of Clinical Endocrinology (ACE/AACE) published similar recommendations, but as initial non-statin therapy included PCSK9 inhibitors along with ezetimibe and recommend bempedoic acid or inclisiran and second line therapy [36]. The 2022 ACC ECDP provided recommendation for incorporating subclinical atherosclerosis imaging with coronary artery calcium (CAC) scoring to better define ASCVD risk and an LDL cholesterol threshold for adding non-statin therapy for primary prevention in adults aged 40 to 75 without diabetes and whose LDL cholesterol < 190 mg/dL [9]. For individuals with a CAC score *≥* 100 AU or *≥* 75th percentile for age, gender and race on a maximally tolerated statin, ezetimibe is recommended if the LDL cholesterol *≥* 70 mg/dL. The addition of a PCSK9 inhibitor was recommended only if the CAC was very high and *≥* 1000 AU.

There is now accumulating evidence of the benefit of non-statin LDL cholesterol lowering therapy in reducing ASCVD risk in primary prevention. Genetic studies suggest that ezetimibe, bempedoic acid and the PCSK9 inhibitors reduce the risk of ASCVD similar to statins when compared to an equivalent degree of LDL cholesterol reduction. In CLEAR Outcomes bempedoic acid significantly reduced the risk of a CV event in high-risk primary prevention patients who were statin intolerant. Hence it may be less important which non-statin add-on therapy is chosen, but more important to achieve the patient’s recommended LDL cholesterol goal based on their underlying CV risk. Clinicians now have a variety of safe and effective non-statin options for lowering LDL cholesterol (see Table 1). The selection of add-on non-statin therapy should be a shared decision between patient and clinician, prioritizing the use of medications with proven outcomes [48]. When choosing a non-statin LDL cholesterol lowering therapy, the clinician should consider factors such as medication preference, potential adverse effects, required LDL cholesterol reduction, and medication accessibility and cost. Overcoming clinician inertia is crucial to optimizing LDL cholesterol management [49]. The availability of several safe and effective non-statin options provides a valuable opportunity to achieve LDL cholesterol goals in a greater proportion of high-risk primary prevention individuals, reducing the burden of ASCVD in the patients we care for.

**Table 1:** Non-statin therapies available to lower LDL cholesterol in primary prevention

Non-statin medication	Mechanism of Action	Doses	Mean percentage LDL-C reduction	Adverse Effects	Primary Prevention Clinical Trial
Ezetimibe	Reduces cholesterol absorption in the small intestine through inhibition of Niemann-Pick C1-like 1 protein	Orally10 mg daily	As monotherapy LDL-C reduced by 18%, and by 25% in combination therapy with statin therapy	Upper respiratory tract infection, diarrhea, arthralgia, sinusitis, pain in extremities	EWTOPIA-75 assessed the efficacy of ezetimibe without statin therapy for reducing ASCVD events in 3796 patients 75 years of age or older who had no history of cardiovascular disease. All participants were randomized to receive either ezetimibe or usual care. Over a median follow-up of 4.1 years, ezetimibe significantly reduced the incidence of the primary outcome (composite of sudden cardiac death, myocardial infarction, coronary revascularization, or stroke) by 34% (hazard ratio 0.66, 95% CI, 0.50–0.86; *p* = 0.002)
PCSK9 mAb Alirocumab	Monoclonal antibodies attach to PCSK9, leading to an increase in LDL receptors, which subsequently removes LDL-C from the bloodstream	Subcutaneously self-administered: available in 2 doses, 75 mg or 150 mg given subcutaneously every 2 weeks.	Alirocumab 75 mg and 150 mg every 2 weeks reduced LDL-C by an additional 45% and 58%, respectively when added to maximally tolerated statin therapy	Nasopharyngitis, injection site reactions, influenza	
PCSK9 mAb Evolocumab	Monoclonal antibodies attach to PCSK9, leading to an increase in LDL receptors, which subsequently removes LDL-C from the bloodstream	Subcutaneously self-administered 140 mg every 2 weeks or 420 mg once monthly (420 mg dose not routinely available and only for patients with latex allergy)	Evolocumab 140 mg every 2 weeks and 420 mg SC every 4 weeks reduces LDL-C by an additional 64% and 58%, respectively when added to maximally tolerated statin	Nasopharyngitis, injection site reactions, influenza The prefilled 140 mg syringe contains latex and is contraindicated in patients with a latex allergy	VESALIUS-CV trial is a randomized, double-blind, placebo-controlled study designed to assess the impact of evolocumab on major adverse cardiovascular events in high-risk patients without prior ischemic events. The study enrolled 12,301 participants with either established atherosclerosis or high-risk diabetes, but no prior myocardial infarction or stroke. Randomization was conducted in a 1:1 ratio, assigning participants to either evolocumab 140 mg administered subcutaneously every 2 weeks or a matching placebo. The primary efficacy objective is to determine if evolocumab reduces the incidence of the co-primary composite endpoint of either coronary heart death, myocardial infarction, or ischemic stroke. This trial is currently ongoing
Inclisiran	siRNA targeting PCSK9, inhibition of PCSK9 translation through RNA interference leads to decreased degradation of LDL receptors, resulting in enhanced clearance of LDL cholesterol	Subcutaneously 284 mg on day 1, day 90, and then every 6 months.	Reduces LDL-C by 48 to 52% when added to maximally tolerated statin	Injection site reaction, arthralgia, urinary tract infection	VICTORION-1 PREVENT is an ongoing clinical trial, targeting to enroll 14,000 participants to evaluate the effect of inclisiran sodium 300 mg s.c. (equivalent to 284 mg inclisiran) compared to placebo on reducing the risk of major CV events in adult patients at high risk for their first major adverse cardiovascular event
Bempedoic acid	Adenosine triphosphate-citrate lyase inhibitor which leads to reduction in cholesterol synthesis and promotes upregulation of LDL receptor expression, leading to enhanced clearance of LDL particles from the circulation.	Orally: 180 mg daily bempedoic acid is also available as a fixed-dose combination with ezetimibe (180 mg bempedoic acid combined with 10 mg ezetimibe	Reduces LDL-C by 17 to 18% when combined with statins Bempedoic acid when combined with ezetimibe reduces LDL-C by 36%	Hyperuricemia, tendon rupture, elevated liver enzymes, abdominal pain, back pain	The CLEAR-Outcomes trial enrolled over 4000 high-risk primary prevention patients to either bempedoic acid or placebo. In a pre-specified subgroup analysis of these primary prevention patients, bempedoic acid significantly reduced LDL-C by 21.3%. At a median follow-up of 39.9 months, the incidence of the composite primary endpoint (CV death, nonfatal myocardial infarction, nonfatal stroke, or coronary revascularization) was lower in the bempedoic acid group (5.3%) compared to placebo (7.6%), (hazard ratio 0.68, 95% CI 0.53–1.87).
Bile acid sequestrants	Bind bile acids causing upregulation of cholesterol conversion into bile acids by the liver	Colesevalam: 6 tablets orally once daily or 3 tablets orally twice daily Cholestyramine 8–16 mg daily in 2 divided doses	Colesevalam as monotherapy reduces LDL by 15% Cholestyramine as monotherapy reduces LDL-C by 10.4%	Constipation, dyspepsia, and nausea, worsening hypertriglyceridemia	The LRC-CPPT is a randomized, double-blind trial that investigated the effects of cholestyramine in asymptomatic men with primary hypercholesterolemia (LDL cholesterol *≥* 190 mg/dL). Over an average follow-up of 7.4 years, 3,806 participants were assigned to receive either cholestyramine or placebo. The study found a significant reduction in the cumulative incidence of the primary endpoint (defined as definite coronary heart disease death and/or definite nonfatal myocardial infarction). The cholestyramine group experienced a 7% event rate compared to 8.6% in the placebo group (hazard ratio 0.81, 95% CI, *p* < 0.05).

ASCVD = Atherosclerotic Cardiovascular Disease; CLEAR Outcomes = Evaluation of Major Cardiovascular Events in Patients with or at high risk for cardiovascular disease who are statin intolerant with Bempedoic Acid or Placebo; CV = cardiovascular; EWTOPIA 75 = Ezetimibe Lipid-Lowering Trial on Prevention of Atherosclerotic Cardiovascular Disease in patients aged 75 years or older; LDL-C = Low Density Lipoprotein Cholesterol; LRC-CPPT = Lipid Research Clinics Coronary Primary Prevention Trial; mAb = monoclonal antibody; PCSK9 = proprotein convertase subtilisin/kexin type 9;RNA = Ribonucleic acid; siRNA = synthetic small interfering ribonucleic acid; SC = subcutaneous; VESALIUS -CV = A Double-blind, Randomized, Placebo-controlled, Multicenter Study to Evaluate the Impact of Evolocumab on Major Cardiovascular Events in Patients at High Cardiovascular Risk Without Prior Myocardial Infarction or Stroke; VICTORION-1Prevent = A Randomized, Double-blind, Placebo-controlled, Multicenter Trial, Assessing the Impact of Inclisiran on Major Adverse Cardiovascular Events in Participants at high risk of a first major cardiovascular event. Adapted from Table 3 of the 2022 ACC Expert Consensus Decision Pathway on the Role of Non-statin Therapies for LDL Cholesterol Lowering in the Management of Atherosclerotic Cardiovascular Disease Risk and from primary prevention outcome trials of non-statin therapies for the primary prevention of ASCVD [9, 14, 20, 29, 34]

### Key References

• • Lloyd-Jones DM, Morris PB, Ballantyne CM, et al.: 2022 ACC Expert Consensus Decision Pathway on the Role of Nonstatin Therapies for LDL-Cholesterol Lowering in the Management of Atherosclerotic Cardiovascular Disease Risk. Journal of the American College of Cardiology 2022, 80(14):1366–1418. 10.1016/j.jacc.2022.07.006. **The newest recommendations from the American College of Cardiology for selecting non-statin therapies in high risk patients on a maximally tolerated statin who need additional LDL cholesterol lowering.**
• Visseren FLJ, Mach F, Smulders YM, et al.: 2021 ESC Guidelines on cardiovascular disease prevention in clinical practice. European Heart Journal 2021, 42(34):3227–3337. 10.1093/eurheartj/ehab484. **Newest European recommendations for adding non-statin therapies for high risk patients.**
• Nissen SE, Lincoff AM, Brennan D, et al.: Bempedoic Acid and Cardiovascular Outcomes in Statin-Intolerant Patients. New England Journal of Medicine 2023, 388(15):1353–1364. 10.1056/NEJMoa2215024. **This randomized trial evaluated the efficacy and safety of bempedoic acid compared to placebo in statin-intolerant patients with or at high risk for cardiovascular disease. The study demonstrated that bempedoic acid significantly reduced LDL cholesterol levels and lowered the risk of cardiovascular events.**
• Nissen SE, Menon V, Nicholls SJ, et al.: Bempedoic Acid for Primary Prevention of Cardiovascular Events in Statin-Intolerant Patients. JAMA 2023, 330(2):131–140. 10.1001/jama.2023.9696. **A subgroup analysis of the original study**, **demonstrating that in high risk primary prevention population**, **bempedoic acid therapy was associated with a significant reduction in major adverse cardiovascular events.**

## Data Availability

No datasets were generated or analysed during the current study.
